# Storage Quality Improvement of Duck Breast Meat: Role of Ultrasound-Assisted Slightly Acidic Electrolyzed Water

**DOI:** 10.3390/foods14030534

**Published:** 2025-02-06

**Authors:** Anqi Xu, Siyi Zhang, Chuanming Huan, Sumin Gao, Hengpeng Wang, Ziwu Gao, Ruiyun Wu, Zhenyu Wang, Xiangren Meng

**Affiliations:** 1 Key Laboratory of Chinese Cuisine Intangible Cultural Heritage Technology Inheritance, Ministry of Culture and Tourism, College of Tourism and Culinary Science, Yangzhou University, Yangzhou 225127, China; 17320013036@163.com (A.X.); 13218949058@163.com (S.Z.); huanchuanming@gmail.com (C.H.); gsumin@163.com (S.G.); yzuwhp@163.com (H.W.); 2 Key Laboratory of Agro-Products Processing, Ministry of Agriculture and Rural Affairs, Beijing 100193, China; wuruiyun814@163.com (R.W.); gzw96530@163.com (Z.G.); 3 Laboratory of Processing Technology Integration for Chinese-Style Meat and Vegetable Dishes, Ministry of Agriculture and Rural Affairs, Institute of Food Science and Technology, Chinese Academy of Agricultural Sciences, Beijing 100193, China; 4 School of Food and Engineering, Yangzhou University, Yangzhou 225127, China

**Keywords:** fresh duck meat, ultrasound, slightly acidic electrolyzed water, high-throughput sequencing, preservation of freshness

## Abstract

This study evaluated the effects of ultrasound-assisted slightly acidic electrolyzed water (SAEW) treatment on duck breast meat storage quality. The impact of different treatments—ultrasound combined with SAEW, SAEW alone, ultrasound combined with water, and water-alone treatment—on the freshness, texture, protein oxidation, and microbiological diversity of the meat was assessed under vacuum packaging at 4 °C. The results demonstrated that the ultrasound–SAEW combination significantly slowed pH decline, inhibited total volatile basic nitrogen (TVB-N) formation, and preserved the redness (a*) and texture of duck breast meat. Additionally, carbonyl and total sulfhydryl measurements indicated that the combined treatment delayed protein oxidation. 16S rDNA analysis showed that combined treatment reduced the microbial abundance, particularly *Pseudomonas* and *Candida*. After 9 days of storage, the total viable count (TVC) of the treated duck breast meat remained within the GB 16869—2005 microbial contamination threshold for fresh meat (5.56 log_10_ CFU/g). These findings highlight the effectiveness of ultrasound-assisted SAEW in extending the shelf life and maintaining the quality of duck breast meat.

## 1. Introduction

Duck meat, richly abundant in superior proteins, unsaturated fatty acids, vitamins, and minerals, is an essential nutritional source with various health benefits, including promoting muscle growth, improving cardiovascular health, and enhancing immune function. It has increasingly gained popularity among consumers, becoming a top choice among meat offerings in the market [1]. However, duck meat is highly susceptible to microbial contamination and enzymatic reactions during storage and transportation, leading to quality deterioration, such as color changes, flavor degradation, and texture softening [2]. These issues have significantly limited its market competitiveness and consumer appeal. Although conventional techniques like cold storage and vacuum packaging could somewhat prolong the freshness of duck meat, they failed to effectively inhibit microbial growth and lipid oxidation, resulting in limited preservation efficacy [3]. Consequently, more effective preservation technologies are urgently needed to prolong the freshness of duck meat while maintaining its high-quality attributes.

Ultrasound-assisted SAEW technology, as an innovative food preservation strategy, combines the cavitation effects of ultrasound with the chemical activity of SAEW to disrupt microbial cells synergistically [4]. Specifically, the high-pressure microbubbles generated by ultrasound in liquid media damage microbial cell structures, causing membrane rupture and leakage of intracellular contents, thereby effectively inhibiting microbial growth and reproduction [5]. Additionally, reactive chlorine and oxygen species in SAEW further oxidize microbial proteins and nucleic acids, achieving efficient sterilization [6,7].

The synergistic action of ultrasound and SAEW offers distinct advantages in food preservation [8]. Evidence indicates that combined ultrasound and SAEW treatment significantly reduced Enterobacteriaceae and mesophilic bacteria populations in chicken breast meat without promoting lipid or protein oxidation [9] and effectively delayed beef quality deterioration, extending its shelf life to 8 days [10]. Moreover, this technology successfully slowed the increases in TVC, *Pseudomonadaceae*, and hydrogen sulfide-producing bacteria, thereby preserving sea bass’s freshness, flavor, and texture [11]. Despite the recognized potential of ultrasound-assisted SAEW technology in preserving various food products, research on its application to duck meat remains limited, particularly concerning systematic evaluations.

The research concentrated on the impact of ultrasound-assisted SAEW treatment on the storage quality of duck breast meat. Key indicators, including freshness, texture properties, protein oxidation, and microbial diversity, were systematically monitored during storage to elucidate the preservation mechanisms of this technology. The findings aim to provide the meat processing industry with a more efficient and safe preservation method, extend the shelf life of meat products, enhance overall product quality, and improve consumer satisfaction. Ultimately, this study aims to foster advancements and sustainable practices within the meat processing sector.

## 2. Materials and Methods

### 2.1. Materials and Chemicals

The 38-day-old skinless Cherry Valley duck breast meat was supplied by Hebei Dongfeng Breeding Co., Ltd., Cangzhou, Hebei, China. Slightly acidic electrolyzed water (SAEW) was provided by Yantai Fangxin Water Treatment Co., Ltd., Yantai, Shandong, China. Analytical-grade reagents, including sodium chloride, potassium chloride, boric acid, hydrochloric acid, potassium carbonate, anhydrous ethanol, ethyl acetate, trichloroacetic acid, and guanidine hydrochloride, were purchased from Sinopharm Chemical Reagent Co., Ltd., Bejing, China.

### 2.2. Sample Preparation

In this research, the SAEW was generated by passing a 5% NaCl solution through an ion-exchange membrane in an oxidation–reduction potential water generator. Prior to utilization, the available chlorine concentration (ACC) was quantified with a chlorine meter, while the pH and oxidation–reduction potential (ORP) were assessed using a pH/ORP meter. The SAEW had a pH of 6.07 ± 0.09, an ORP of 862.0 ± 5.0 mV, and an ACC of 28.83 ± 1.47 mg/L.

After slaughter, duck meat was sealed in vacuum packaging and swiftly shipped to the laboratory within 2 h to maintain its freshness. The meat was then chilled with ice packs to ensure its quality before undergoing further sterilization procedures. Duck breast meat soaked in pure water for 15 min was designated as the WD group. Duck breast meat soaked in SAEW for 15 min was called the SD group. Duck breast meat treated with ultrasound (200 W, 28 kHz) in pure water for 15 min was labeled as the UWD group. Finally, duck breast meat treated with ultrasound (200 W, 28 kHz) in SAEW for 15 min was categorized as the USD group. The duck breast meat-to-water or SAEW ratio was maintained at 1:5 in all treatments. Additionally, during ultrasonic treatments, the duck breast meat-to-water or SAEW ratio was maintained at 1:5 across all groups. Specifically, 200 g ± 2.87 g of duck breast meat was immersed in 1000 g ± 1.04 g of pure water or SAEW. Ultrasonic treatment was conducted using an ultrasonic transducer (RC-1000LG, Renchuan Technology Co., Ltd., Langfang, China; internal dimensions: 350 × 300 × 200 mm), ensuring uniform exposure to ultrasonic waves. The samples were sealed in polyethylene bags during treatment to prevent evaporation and maintain consistent treatment conditions.

The ultrasonic power of 200 W and frequency of 28 kHz were specifically chosen to optimize cavitation and energy transfer within the meat matrix while avoiding undesirable effects such as overheating or structural damage. These parameters were based on prior research indicating that low-frequency ultrasound (20–100 kHz) enhances heat and mass transfer while preserving the sensory and nutritional quality of food products [12]. Additionally, our preliminary experiments confirmed that this combination effectively balanced microbial inactivation with sensory quality preservation. The treatment duration of 15 min was selected following preliminary experiments, which demonstrated that this duration achieved significant improvements in storage quality without negatively impacting the meat’s structural or sensory attributes. Shorter treatment durations were insufficient, while longer durations provided no added benefits.

After treatment, all samples were enclosed in polyethylene packaging and stored at 4 °C. Analyses were performed every 6 days over a 12-day storage period to evaluate the effects of the various treatments on the duck breast meat’s storage quality.

### 2.3. Color Analysis

The L*, a*, and b* values of duck breast meat samples were assessed using a colorimeter at three selected sites on each sample. Each measurement was repeated five times per sample. Before measurement, the colorimeter was calibrated with a whiteboard to ensure accuracy.

### 2.4. pH Analysis

Following the Chinese National Standard GB 5009.237-2016 [13], duck breast meat (1 g) was blended with 10 mL of 0.1 mol/L KCl solution, homogenized, and then analyzed. The electrode was immersed in the homogenate, and measurements were taken once the readings stabilized, with each sample assessed thrice.

### 2.5. Determination of TVB-N

The TVB-N content was ascertained via the microdiffusion technique, adhering to the GB/T 5009.228-2016 protocol [14]. A 20 g sample of duck breast meat was mixed with 100.0 mL water, stirred, and macerated for 30 min before filtration. A drop of boric acid solution containing an indicator was placed in the diffusion dish’s center, with the periphery filled with the sample solution and saturated potassium carbonate. The mixture was well mixed and incubated at 37 °C ± 1 °C for 2 h. Post-incubation, the solution underwent titration with 0.01 mol/L HCl. The titration results were computed using Formula (1), reporting values in mg N/100 g.

(1) $$X=\frac{(V_{1}-V_{2})\times c\times 14}{m\times (V/V_{0})}\times 100$$

where *X* is the content of TVB-N in the sample (mg/100 g); *V*_1_ is the volume of HCl or H_2_SO_4_ standard titration solution consumed by the sample solution (mL); *V*_2_ is the volume of HCl or H_2_SO_4_ standard titration solution consumed by the reagent blank (mL); *c* is the concentration of the HCl or H_2_SO_4_ titration solution in mol/L; 14 is the mass of nitrogen (g/mol) corresponding to titration with 1.0 mL of standard HCl or H_2_SO_4_; m is the mass of the sample (g); *V* is the accurately measured volume of the filtrate (mL), where *V* = 1 in this method; *V*_0_ is the total volume of the sample solution (mL), where *V*_0_ = 100 in this method; and 100 is the conversion factor to express the results as mg/100 g.

### 2.6. Textural Properties Analysis (TPA)

The methodology was adapted from previous studies with slight modifications [15]. To ensure uniformity and precision, the duck breast meat was cut into cubes with dimensions of 1 cm × 1 cm × 1 cm using a metal mold designed explicitly for this purpose. Springiness, chewiness, and hardness were measured using a TMS-Pro/Touch texture analyzer. The probe model was SMS P/50, and the testing mode was TPA. The testing parameters were as follows: pre-test speed was 2 mm/s, test speed was 1 mm/s, post-test speed was 5 mm/s, compression degree was 35%, number of compressions was 2, compression interval was 5 s, trigger type was automatic, and trigger force was 5 g. The texture analysis was conducted along the muscle fibers to ensure consistency in the measurements. Each group consisted of nine parallel samples, and the results were averaged.

### 2.7. Determination of Microstructure

Duck breast meat was cut into dimensions of 2.5 × 2.5 × 2 mm^3^ along both the vertical and parallel directions of the muscle fibers. The samples were placed in a 4% tissue fixative solution and fixed at 4 °C for 12 h. After fixation, the samples were subjected to tissue dehydration, embedding, sectioning, baking, hematoxylin–eosin (HE) staining, and mounting. Cross-sectional slices were observed under optical microscopes at 400× magnifications.

### 2.8. Determination of the Content of Carbonyl

The carbonyl content was determined with slight modifications based on previous methods [16]. Myofibrillar protein solution from duck breast meat was mixed with 10% trichloroacetic acid (TCA) and centrifuged. After removing the supernatant, the precipitate was treated with 2,4-dinitrophenylhydrazine (DNPH) solution and incubated in the dark at 25 °C for 1 h. Subsequently, the mixture was combined with 20% TCA and centrifuged (10,000× *g*, 5 min, 4 °C) to remove the supernatant. The precipitate was washed with 1 mL of an ethanol–ethyl acetate solution (1:1, *v*/*v*) to eliminate unreacted DNPH. After washing, 1 mL of 6 mol/L guanidine hydrochloride solution was added to the precipitate.

The mixture was incubated at 37 °C for 30 min and centrifuged (10,000× *g*, 3 min, 25 °C) to collect the supernatant. The absorbance of the supernatant was measured at 370 nm. Each sample was tested in triplicate, and the carbonyl content was calculated using Formula (2).

(2) $$Carbonyl\;content\;(\mathrm{nmol}/g\;\mathrm{protein})=\frac{A_{412}}{\left(\varepsilon \times C\right)}$$

ε is the molar extinction coefficient (22,000 mol^−1^·cm^−1^) and *C* is the concentration of the myofibrillar protein being measured.

### 2.9. Determination of the Content of Total Sulfhydryl

The total sulfhydryl content was determined with slight modifications to previous methods [17]. A total of 0.5 mL of myofibrillar protein solution from duck breast meat (4 mg/mL) was mixed with 4.5 mL Tris-HCl buffer (0.2 M Tris, 8 M urea, 2% SDS, 10 mM EDTA). The mixture was vortexed thoroughly, and 1 mL of the solution was discarded. Subsequently, 0.4 mL of DTNB (0.1%) was added to the remaining mixture and mixed well. The solution was incubated in a water bath at a constant temperature of 40 °C for 25 min, and the absorbance was measured at 412 nm. Each sample was tested in triplicate, and the total sulfhydryl content was calculated using Formula (3).

(3) $$Total\;sulfhydryl\;content\;(µ\mathrm{mol}/g\;\mathrm{protein})=[\frac{A_{412}}{\left(\varepsilon \times C\right)}]\times 10^{6}$$

ε is the molar extinction coefficient (22,000 mol^−1^·cm^−1^) and *C* is the concentration of the myofibrillar protein being measured.

### 2.10. Determination of Total Vaible Count (TVC)

The TVC was determined using the standard plate count method, following the Chinese National Standard GB 4789.2 (2010) [18]. An amount of 10 g of duck breast meat was mixed with 90 mL of sterile saline and homogenized at 8000 rpm for 1 min. The resulting homogenate was serially diluted tenfold in sterile saline. A total of 1 mL of the appropriately diluted sample was placed onto plate count agar. The plates were incubated in a constant-temperature incubator at 37 ± 2 °C for 48 ± 2 h. Colony counts were performed at 37 ± 2 °C, and the results were expressed as log10 CFU/g.

### 2.11. Analysis of Microbial Diversity of Duck Breast Meat Bacteria During Storage

The genomic DNA of bacteria and fungi from duck breast meat was extracted using a DNA extraction kit. The bacterial 16S rDNA V3-V4 region was amplified with the primers 338F-806R (338F 5′-ACTCCTACGGGAGGCAGCAG-3′ and 806R 5′-CCGTCAATTCMTTTRAGTTT-3′), generating a 480 bp fragment. The standard eukaryotic ITS1 region was amplified using the primers ITS1F-ITS2 (ITS1F 5′-CTTGGTCATTTAGAGGAAGTAA-3′ and ITS2 5′-GCTGCGTTCTTCATCGATGC-3′), producing a 250 bp fragment. Sequencing was performed using the NovaSeq-PE250 strategy.

Raw sequencing data obtained from 16S rDNA analysis were initially screened based on sequence quality. Problematic samples were re-sequenced or supplemented as needed. 16S rDNA analysis was demultiplexed according to index and barcode information, and barcode sequences were removed. Sequence denoising or operational taxonomic unit (OTU) clustering was conducted using the QIIME2 DADA2 pipeline and the Vsearch workflow (https://docs.qiime2.org/2024.10/) (accessed on 15 January 2025).

The taxonomic composition of each sample (group) at different taxonomic levels was analyzed to provide an overview of the microbial community. The α-diversity of each sample was evaluated based on the distribution of ASVs/OTUs across samples, and rarefaction curves were used to assess whether sequencing depth was sufficient. At the taxonomic composition level, unsupervised and supervised ordination, clustering, and modeling methods were applied and combined with appropriate statistical tests to further evaluate differences in species abundance among different groups and identify potential biomarker species (https://www.genescloud.cn/home) (accessed on 15 January 2025).

### 2.12. Statistical Analysis

Experiments were conducted in triplicate, with results presented as mean ± SD. Duncan’s test was applied for multiple comparisons at *α* = 0.05. An ANOVA was utilized for assessing differences among treatments with SPSS 23.0, while graphs and charts were created using Origin 2024 and GraphPad Prism 9.

## 3. Results

### 3.1. Analysis of the Effect of Ultrasound-Assisted SAEW on the Freshness of Duck Breast Meat

#### 3.1.1. Changes in pH of Duck Breast Meat During Storage

**Figure 1:**
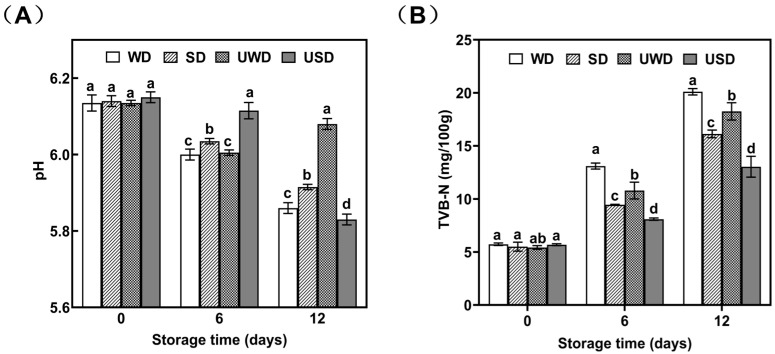
Changes in pH of duck breast during storage (**A**); changes in TVB-N of duck breast during storage (**B**). WD: duck breast meat treated with pure water; SD: duck breast meat treated with SAEW; UWD: duck breast meat treated with ultrasound combined with pure water; USD: duck breast meat treated with ultrasound combined with SAEW. Different letters indicate statistically significant differences at a level of *p* < 0.05.

The pH level is essential in assessing meat quality, as its value is closely associated with freshness. Figure 1A depicted the pH fluctuations in duck breast meat subjected to various treatments throughout cold storage. On day 0, the initial pH values of all treatment groups ranged from 6.13 to 6.20, consistent with the typical pH range of fresh meat (5.8–6.2) [19], indicating that the duck breast meat was in a relatively high-quality state.

Over the first six days of storage, all groups experienced a pH decrease likely due to microbial fermentation leading to organic acid accumulation in the meat. As storage progressed, nitrogenous compounds in the duck breast meat were broken down by microbial and enzymatic activity into amino acids, ammonia, indoles, and other basic substances [20]. This process mitigated the pH decrease, indicating a gradual decline in meat quality.

By day 12, the UWD group had the highest pH, and the USD group had the lowest. The respective pH values for the WD, SD, UWD, and USD groups were 5.86, 5.91, 6.08, and 5.83. The USD group’s pH was markedly lower compared to other groups with statistical significance (*p* < 0.05). The findings suggest that the dual application of ultrasound and slightly acidic electrolyzed water effectively suppressed spoilage bacteria in duck breast meat, thereby delaying pH rise and limiting the formation of basic nitrogenous compounds. This treatment showed beneficial effects on maintaining the quality of duck breast meat.

#### 3.1.2. Changes in Color of Duck Breast Meat During Storage

Color is a critical factor influencing the consumer perception of meat freshness and acceptance. Among color parameters, the a* value is the most critical indicator of fresh meat color, with higher a* values indicating a brighter red color, which is more appealing to consumers [21]. The color of duck meat was closely related to the state and content of myoglobin. Fresh duck meat contained more ferrous myoglobin (Mb−Fe^2+^), resulting in higher a* values. Over time, Mb−Fe^2+^ was oxidized to ferric myoglobin (Mb−Fe^3+^), causing the color to shift towards brown and the a* value to decrease [22].

As shown in Table 1, the a* values of all treatment groups gradually declined during refrigerated storage, reaching their lowest levels by day 12. In the early storage stages (0–6 days), no significant differences in a* values were observed among the groups (*p* < 0.05). By day 12, the a* value for the UWD group was significantly lower than other groups (*p* < 0.05). This decrease might be due to free radicals and thermal and acoustic effects from ultrasound, which can damage muscle cell pigment structures [23]. Conversely, the a* value of the USD group was markedly higher than the UWD group (*p* < 0.05). This is likely due to the protective effects of ions in SAEW (Cl^−^, Na^+^, ClO^−^, and HClO), which preserved muscle cell integrity and inhibited oxidative reactions [24]. Additionally, the L* and b* values of all groups exhibited fluctuations during storage without a clear pattern of change.

In summary, different sterilization methods significantly affected the a* of duck breast meat. Ultrasound treatment negatively impacted the maintenance of a*, while SAEW played a positive role in preserving the a* value.

#### 3.1.3. Changes in TVB-N of Duck Breast Meat During Storage

TVB-N is a key indicator of meat freshness and quality. TVB-N mainly comprises ammonia and volatile amines, including primary, secondary, and tertiary amines, resulting from the breakdown of nitrogenous compounds like proteins and amino acids in meat [25].

Figure 1B displays TVB-N values for duck breast meat across various treatments. Initially, all groups had low TVB-N levels, signifying high freshness. As storage progressed, microbial activity led to the breakdown of proteins into volatile nitrogenous compounds, increasing TVB-N levels across all groups [26]. By day 12, the TVB-N levels for the WD, SD, UWD, and USD groups rose to 20.1 mg/100 g, 16.13 mg/100 g, 18.25 mg/100 g, and 13.04 mg/100 g, respectively, marking increases of 250.79%, 193.27%, 236.1%, and 129.17% from their starting values.

The SD and USD groups showed significantly lower rates of TVB-N increase compared to the other groups (*p* < 0.05). This was likely due to the slightly acidic nature of SAEW, which reduced the activity of most alkaline bacteria and partially inhibited the synthesis of basic nitrogenous compounds [27]. Furthermore, the high pressure generated by microbubbles oscillating in the ultrasonic field caused microbial cell membranes to rupture, allowing SAEW to penetrate the cells and accelerate cell death [28]. The findings suggest that the ultrasound and SAEW combination effectively delayed TVB-N formation in duck breast meat during cold storage, preserving its freshness.

### 3.2. Analysis of the Effect of Ultrasound-Assisted SAEW on Texture of Duck Breast Meat

#### 3.2.1. Changes in TPA of Duck Breast Meat During Storage

**Figure 2:**
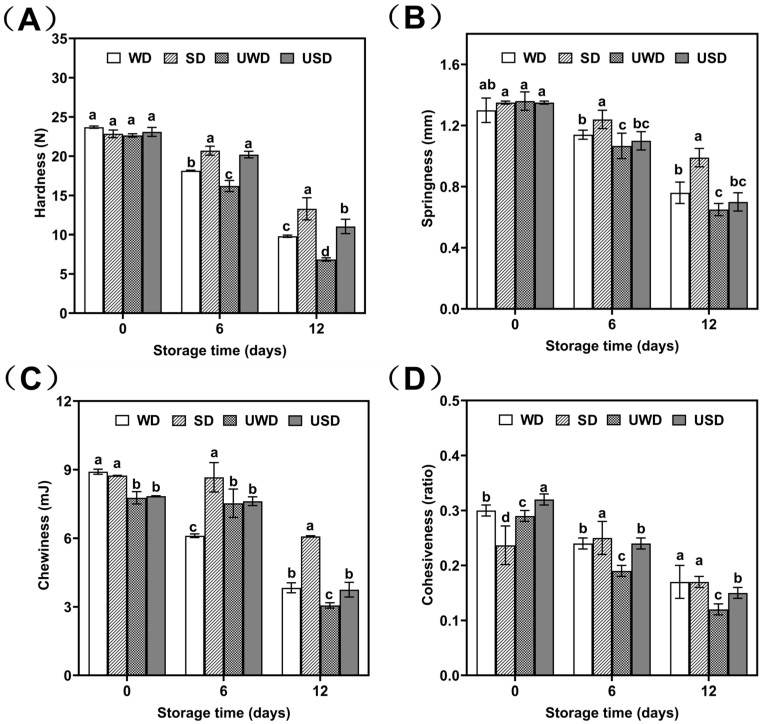
Changes in TPA of duck breast meat during storage. (**A**) Hardness (N); (**B**) Springiness (mm); (**C**) Chewiness (mJ); (**D**) Cohesiveness (ratio). WD: duck breast meat treated with pure water; SD: duck breast meat treated with SAEW; UWD: duck breast meat treated with ultrasound combined with pure water; USD: duck breast meat treated with ultrasound combined with SAEW. The values 0, 6, and 12 represent storage time (days). Different letters within the same column on same day indicate statistically significant differences at a level of *p* < 0.05.

Texture is a critical parameter for evaluating meat product quality, as it reflects freshness and directly impacts consumer satisfaction. As shown in Figure 2, hardness is an important metric describing meat texture. It refers to the force required to deform the meat, representing the internal cohesive force maintaining its structural integrity. Sensory-wise, hardness is perceived as a firm or soft texture, significantly influencing the duck breast meat’s taste and flavor.

By day 12 of storage, the hardness of the duck breast meat decreased by 59.25%, 41.79%, 69.76%, and 52.16% in the WD, SD, UWD, and USD groups, respectively. The UWD group exhibited the lowest hardness value (6.85 N), while the SD group had the highest (13.3 N). The reduction in hardness was attributed to protein cross-linking, denaturation, and degradation during storage. Proteases produced by microorganisms disrupted the structure of myofibrils, leading to fragmentation, which intensified with microbial proliferation [29].

The antimicrobial properties of SAEW reduced microbial growth, allowing duck breast meat to retain freshness and maintain hardness more effectively. Additionally, the sodium chloride in SAEW positively contributed to preserving meat hardness. These findings highlighted the significant role of SAEW in maintaining the structural integrity and textural quality of duck breast meat during storage.

Springiness refers to the ability of a material to deform under an external force and recover its shape once the force is removed. In the initial 0–6 days of storage, the springiness of duck breast meat did not differ significantly among groups, suggesting that ultrasound and SAEW treatments did not adversely affect the meat’s springiness. However, as storage progressed, all samples experienced varying degrees of springiness reduction, with decreases ranging from 27.04% to 52.57%. The trend of springiness reduction followed the order of SD < WD < USD < UWD.

Generally, water content significantly affects meat springiness, with higher water content contributing to more excellent springiness within a certain range [30]. Studies showed that adding sodium tripolyphosphate could reduce water loss and enhance springiness in catfish filets, which was consistent with the results of this study. The slower decrease in springiness observed in the SD group might be attributed to the ionic strength of sodium chloride in SAEW, which promoted the swelling of myofibrillar proteins, improved water retention, and effectively inhibited microbial growth, thereby delaying myofibrillar fragmentation. In contrast, the USD group’s springiness was significantly lower than that of the SD and WD groups. This reduction might be the result of the structural disruption of muscle tissues caused by ultrasound, which increased the fragmentation of myofibrils and subsequently reduced springiness while enhancing tenderness [31].

Additionally, chewiness and cohesiveness decreased across all treatment groups as storage time increased. By day 12, the SD group exhibited significantly lower reductions in chewiness and cohesiveness compared to the WD, UWD, and USD groups (*p* < 0.05). These findings further demonstrate the positive effect of SAEW treatment in preserving the textural properties of duck breast meat.

#### 3.2.2. Analysis of Microstructure

**Figure 3:**
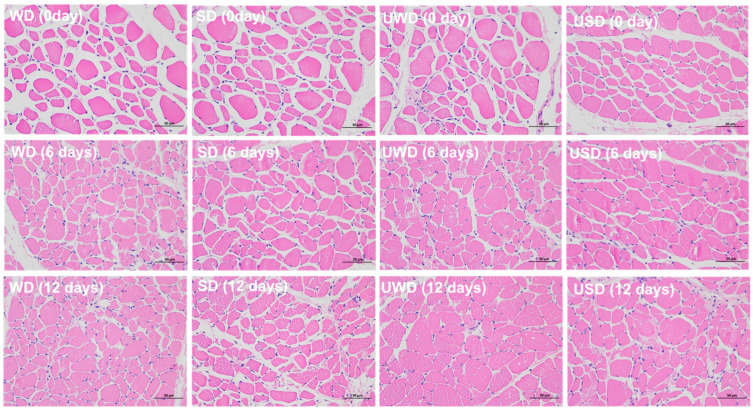
HE staining of duck breast meat. The image was captured using a NIKON Eclipse Ci upright microscope (Nikon Instruments Inc., Tokyo, Japan) with a NIKON Digital Sight DS-FI2 camera system at 400× magnification. The imaging software used was NIS_F_Ver43000_64 bit_E. Scale bar = 50 μm.

During storage, protein denaturation occurs to varying degrees in duck breast meat under different treatments, leading to structural damage. Figure 3 illustrates the alignment of muscle fibers, the integrity of sarcomeres, and the gaps between muscle fibers in duck breast meat from each treatment group during storage. The pink regions represent muscle fibers, while the white gaps indicate the spaces between the fibers. These white gaps reflect structural changes in the muscle tissue during storage, with their size and distribution serving as indicators of the degree of preservation of the muscle structure.

At day 0, the muscle fibers in the WD and SD groups were tightly aligned, with clearly defined sarcomere structures, and minimal white gaps were observed between the fibers. This indicates that SAEW treatment alone had minimal impact on the muscle tissue structure of duck breast meat. In contrast, the UWD and USD groups exhibited larger pink regions and irregular muscle fiber arrangements, with smaller white gaps compared to the WD and SD groups. These irregularities may be attributed to cavitation and turbulent forces caused by ultrasound, which can induce protein fragmentation and localized tissue disruption [32].

As storage progressed, the gaps between muscle fibers (white regions) gradually decreased across all groups, and the sarcomere structures became less defined. A reduction in the size of the white gaps was observed in some groups, indicating structural weakening or degradation of the muscle tissue, which could lead to loosening or disintegration of the tissue over time. By the end of storage, the SD group exhibited the smallest reduction in white gaps and maintained the highest level of sarcomere integrity compared to the other groups. This suggests that SAEW treatment inhibited microbial growth during storage, reducing protein structure degradation caused by microbial metabolism and better preserving the texture of duck breast meat. Additionally, the USD group showed larger white gaps and less sarcomere disintegration compared to the UWD group, suggesting that the combination of ultrasound and SAEW was more effective in preserving the structural integrity of the muscle than ultrasound alone. The relatively larger white gaps in the USD group indicate better maintenance of muscle tissue integrity during storage, further supporting the protective role of SAEW in mitigating structural damage caused by ultrasound treatment.

### 3.3. Analysis of the Effect of Ultrasound-Assisted SAEW on Protein Oxidation of Duck Breast Meat

Sulfhydryls (S-H) are essential functional groups in many proteins and enzyme active sites, but they are highly susceptible to oxidation, forming products such as -SOH, -SOOH, and -SS- [33]. As such, sulfhydryl loss is a crucial marker of protein oxidation. Protein oxidation influences both the nutritional quality and sensory properties of fresh meat, including taste, color, and texture.

**Figure 4:**
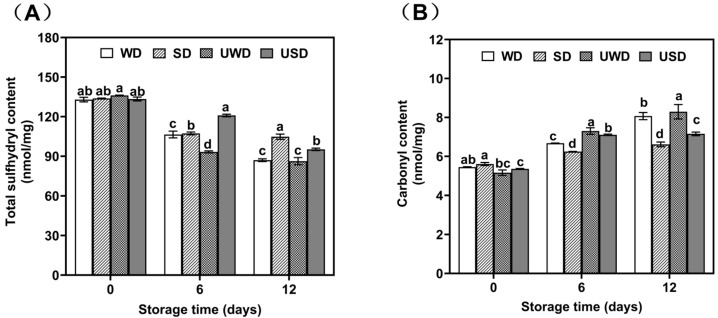
Changes in protein sulfhydryl content of duck breast during storage (**A**); changes in protein carbonyl content of duck breast during storage (**B**). WD: duck breast meat treated with pure water; SD: duck breast meat treated with SAEW; UWD: duck breast meat treated with ultrasound combined with pure water; USD: duck breast meat treated with ultrasound combined with SAEW. Different letters indicate statistically significant differences at a level of *p* < 0.05.

Figure 4A illustrates the total sulfhydryl content of myofibrillar proteins across various treatment groups over the storage period. At the start of storage, initial total sulfhydryl content did not significantly differ among treatment groups. Nevertheless, the total sulfhydryl content in all groups progressively declined over time. The WD and UWD groups exhibited the fastest declines, with sulfhydryl content reductions of 42.85% and 44.18%, respectively, by day 12. In contrast, the SD group showed the slowest decline, with sulfhydryl content decreasing by only 27.29% at the end of storage. The USD group experienced a reduction of 34.97%, lower than the WD and UWD groups but higher than the SD group.

Carbonyls are significant protein oxidation products commonly used to indicate oxidative damage to proteins. Their formation during protein oxidation involves multiple pathways, including the direct oxidation of aliphatic amino acids (e.g., lysine and arginine) into carbonyls, α-amidation leading to β-cleavage of the polypeptide backbone, the Michael addition of unsaturated aldehydes from lipid oxidation, and reactions between lysine and reducing sugars or their oxidation products to form reactive carbonyl derivatives [34].

To further investigate the effects of ultrasound combined with SAEW on protein oxidation in duck breast meat, carbonyl content was measured during storage (Figure 4B). The results showed a gradual increase in carbonyl content across all samples, consistent with the mechanisms of carbonyl formation during protein oxidation. The WD and UWD groups exhibited the most significant increases in carbonyl content (*p* < 0.05). At day 0, the carbonyl content of the WD and UWD groups was 5.45 nmol/g protein and 5.16 nmol/g protein, respectively. By day 6, these values increased to 6.66 nmol/g protein and 7.30 nmol/g protein, and by day 12, they further rose to 8.07 nmol/g protein and 8.29 nmol/g protein. The UWD group showed the highest carbonyl content (8.29 nmol/g protein) at the end of storage.

These results suggest that ultrasound treatment likely promoted excessive protein oxidation through cavitation-induced hydroxyl radical (·OH) production. The chain reactions initiated by free radicals oxidized amino acid side chains, especially aliphatic amino acids like lysine and arginine, directly forming carbonyls or causing β-cleavage of the polypeptide backbone via α-amidation [35].

However, the SD group maintained the lowest carbonyl content throughout storage, and the USD group exhibited significantly lower carbonyl content than the UWD group (*p* < 0.05). A combined analysis of thiol and carbonyl content revealed that SAEW treatment alone effectively delayed protein oxidation in duck breast meat. SAEW inhibited microbial proliferation, reducing the activity of tissue proteases and other oxidative enzymes, thereby slowing the protein oxidation rate [36]. The synergistic effect of SAEW mitigated the excessive protein oxidation induced by ultrasound, helping to preserve the quality of duck breast meat.

### 3.4. Analysis of the Effect of Ultrasound-Assisted SAEW on Microbiology of Duck Breast Meat

#### 3.4.1. Changes in TVC of Duck Breast Meat During Storage

**Figure 5:**
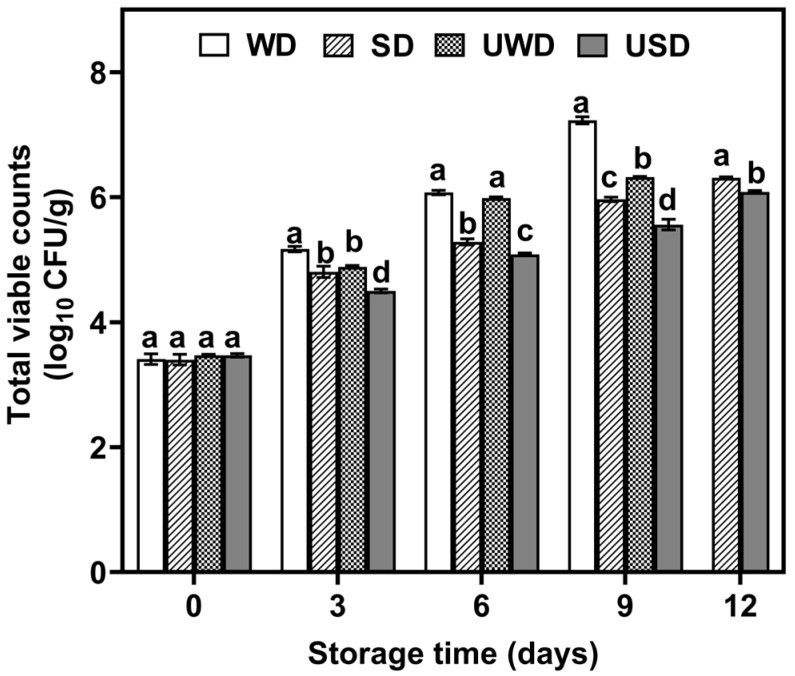
Changes in TVC of duck breast during storage. WD: duck breast meat treated with pure water; SD: duck breast meat treated with SAEW; UWD: duck breast meat treated with ultrasound combined with pure water; USD: duck breast meat treated with ultrasound combined with SAEW. Different letters indicate statistically significant differences at a level of *p* < 0.05.

Microorganism proliferation is the main cause of fresh meat spoilage, with TVC typically needing to stay below 1 × 10^6^ CFU/g. To assess the impact of various sterilization methods on duck breast meat preservation, the TVC for each group was monitored every three days throughout storage. The TVC changes in the treated duck breast meat during storage are shown in Figure 5.

The initial TVC values across all groups were between 3.4 and 3.47 log10 CFU/g, showing no significant differences (*p* < 0.05). As storage time increased, the TVC values in all treatment groups gradually rose (*p* < 0.05). Specifically, the TVC values of the WD, SD, UWD, and USD groups reached the safety threshold of 6.0 log_10_ CFU/g on days 6, 12, 9, and 12, respectively. During storage, the SD and USD groups had significantly lower TVC values compared to the other groups. By day 12, the TVC value in the USD group was 0.23 log_10_ CFU/g lower than that in the SD group, demonstrating superior antimicrobial efficacy.

The enhanced effect might be due to hypochlorous acid (HOCl) in SAEW, which produces hydroxyl (-OH) and chlorine (-Cl) radicals. These radicals can inhibit cytoplasmic enzymes and damage bacterial outer membranes [37]. Moreover, SAEW has a positive oxidation–reduction potential, allowing it to absorb electrons from bacterial cell membranes, leading to membrane instability and facilitating the penetration of antimicrobial agents into the bacteria. This disruption affects bacterial metabolism and ultimately results in bacterial death [38]. Additionally, the cavitation effect of ultrasound can partially damage bacterial cell membranes, enhancing the penetration rate of SAEW into the membranes and thereby boosting sterilization efficacy. These findings suggest a synergistic and amplified antibacterial effect between ultrasound and SAEW.

#### 3.4.2. Changes in Microbial Diversity of Duck Breast Meat During Storage

**Figure 6:**
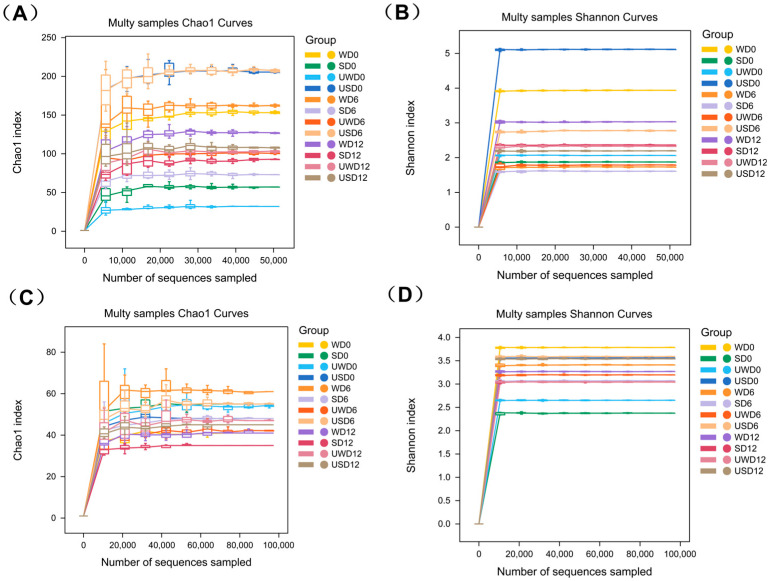
The Chao1 and Shannon curves of bacteria (**A**,**C**) and fungi (**B**,**D**).

Chao 1, Shannon, and Simpson indices were employed to assess the richness and diversity of microbial communities in duck breast meat across various treatments during storage. The Chao 1 index estimates community species richness, with higher values indicating a more significant potential number of species. The Shannon index considers species richness and relative abundance, with higher values reflecting greater community diversity. The Simpson index emphasizes species evenness, where lower values indicate higher evenness within the community [39]. Figure 6 shows that Chao1 rarefaction curves approached the maximum OTU count, indicating varying community richness among samples and sufficient sequencing depth as the curves leveled off. Similarly, Shannon rarefaction curves rose quickly then stabilized, suggesting ample sequencing data for effective analysis.

As shown in Table 2, at the initial storage stage (day 0), the USD group exhibited the lowest Chao 1 and Simpson indices but the highest Shannon index compared to other treatment groups. This result suggests that the USD group had the lowest bacterial species richness and uneven distribution, likely due to the immediate elimination of particular bacterial species by the ultrasound-assisted SAEW treatment, which impacted the initial community richness. As storage progressed, the Chao 1 index and observed species counts in different groups followed distinct trends, likely reflecting microbial community structure changes caused by varying sterilization levels. By the end of the storage period, the USD group maintained relatively low Chao 1 and Shannon indices alongside a high Simpson index. This pattern suggests that ultrasound-assisted SAEW treatment may have inhibited the growth of certain bacteria during storage or promoted the growth of specific species, enabling them to dominate the community.

The changes in fungi alpha diversity during storage are presented in Table 3. Unlike bacterial communities, fungi alpha diversity exhibited different trends. On day 0, the USD group showed the highest Chao 1 index and the lowest Simpson index, indicating more excellent fungi species diversity and evenness in the USD-treated duck breast meat than other groups. However, by the end of the storage period, the USD group exhibited the lowest Chao 1 and Shannon indices and the highest Simpson index. These findings suggest that ultrasound-assisted SAEW treatment might have exerted specific effects on the fungi community or driven dynamic changes in the fungi community structure during storage.

#### 3.4.3. Changes in Microbial Community Composition of Duck Breast Meat During Storage

**Figure 7:**
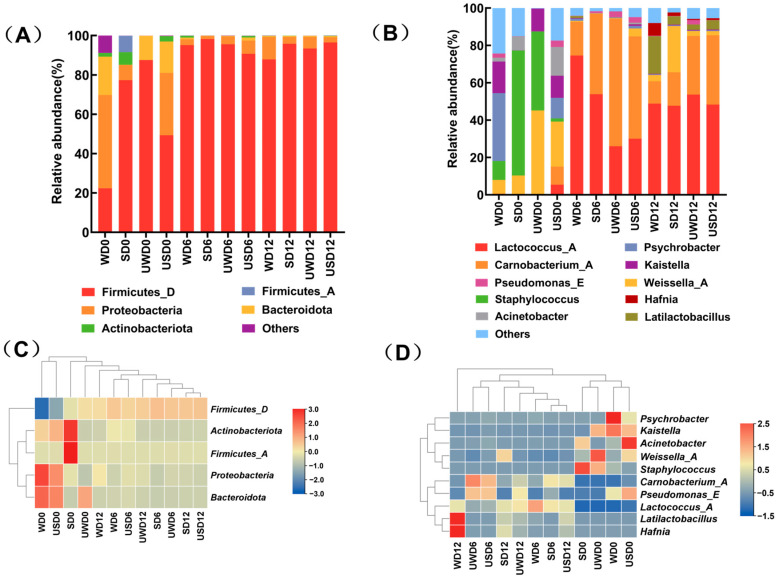
Changes in the bacterial community composition of duck breast during storage. (**A**,**C**) The histogram and clustering heat map at the phylum level. (**B**,**D**) The histogram and clustering heat map at the genus level, showing the top 5 phyla and top 10 genera, respectively.

OTU classification and taxonomic identification allowed for a detailed examination of microbial community composition at various taxonomic levels, including phylum, class, order, family, and genus, for each sample. The microbial community composition of duck breast meat during storage was analyzed at the phylum and genus levels. At the phylum level, 20 phyla and 50 genera were identified across 12 samples (details provided in the Supplementary Materials). Due to the relatively concentrated distribution of microbial species, selecting the top five phyla and top 10 genera ensured that the analysis encompassed the major components of the community. Figure 7A,C illustrates the bacterial composition at the phylum level, focusing on the top five most abundant phyla. The results showed that *Firmicutes*, *Proteobacteria*, and *Actinobacteria* were the dominant phyla. At the initial storage stage (day 0), the abundance of Firmicutes in the USD group was 49.36%, a relatively low level compared to other groups. As storage time progressed, *Firmicutes* and *Proteobacteria* became the dominant phyla in all groups. By day 12, the relative abundances of these phyla reached 99.34%, 99.36%, 99.35%, and 99.22% in the different treatment groups. Notably, the USD group exhibited a lower relative abundance of *Proteobacteria* than other groups, suggesting that ultrasound-assisted SAEW treatment effectively reduced bacterial richness in duck breast meat.

Figure 7B,D further depicts the changes in the top 10 most abundant genera within the bacterial community during storage across treatment groups. Among these genera, *Lactococcus*, *Carnobacterium*, *Weissella*, *Staphylococcus*, and *Latilactobacillus* belonged to the phylum *Firmicutes*, while *Psychrobacter*, *Acinetobacter*, *Pseudomonas*, and *Hafnia* were classified under the phylum *Proteobacteria*. Ultrasound-assisted SAEW treatment significantly reduced the abundances of *Psychrobacter*, *Pseudomonas*, *Kaistella*, *Staphylococcus*, and *Lactococcus*. By storage’s end, the USD group had reduced relative abundances of *Pseudomonas*, *Weissella*, and *Latilactobacillus* compared to other groups.

Notably, *Pseudomonas* fluorescens and *Pseudomonas* putida, which thrive at low temperatures and are known to cause the spoilage of refrigerated foods and produce off-flavors in meat, were among the vital spoilage microorganisms [40,41]. These findings indicate that ultrasound-assisted SAEW treatment significantly inhibited these bacteria, effectively reducing spoilage potential in duck breast meat during storage.

**Figure 8:**
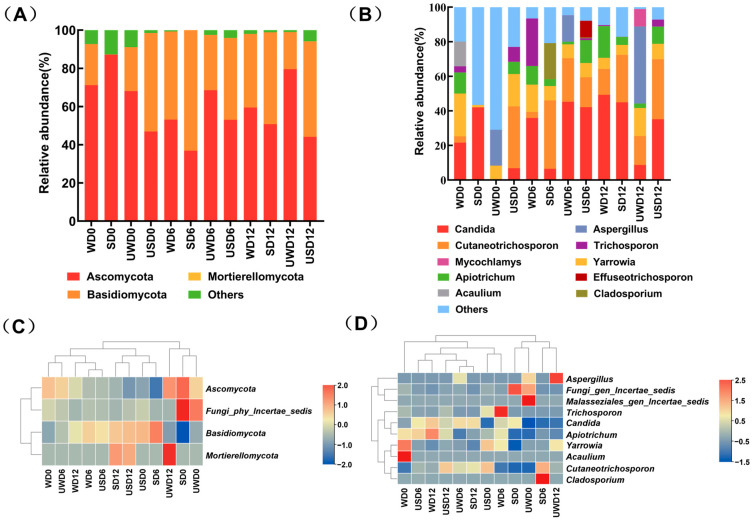
Changes in the fungi community composition of duck breast during storage. (**A**,**C**) The histogram and clustering heat map at the phylum level. (**B**,**D**) The histogram and clustering heat map at the genus level, showing the top 5 phyla and top 10 genera, respectively.

The fungal composition in duck breast meat over storage was examined at the phylum and genus levels. Figure 8A,C depicts the phylum-level fungal composition in duck breast meat, with *Ascomycota* and *Basidiomycota* being the prevalent phyla. Notably, at the initial storage stage (day 0), the abundance of *Ascomycota* in the USD group, treated with ultrasound combined with SAEW, was only 46.95%. This observation suggested that the combined treatment might have inhibited the initial colonization of *Ascomycota*. By day 12 of storage, the abundance of *Ascomycota* in the SAEW-treated groups (SD and USD) was lower than in the non-SAEW-treated groups (WD and UWD). This trend indicated that SAEW significantly inhibited the growth of *Ascomycota*, with ultrasound further enhancing this inhibitory effect.

At the genus level, Figure 8B,D presents the changes in the ten most abundant fungal genera in each treatment group during storage. Among these genera, those belonging to Ascomycota included *Candida*, *Cutaneotrichosporon*, *Yarrowia*, *Aspergillus*, *Cladosporium*, *Effuseotrichosporon*, and *Acaulium*. Genera from *Basidiomycota* included *Trichosporon*. The combined ultrasound and SAEW treatment reduced the abundance of *Candida* and *Acaulium*. As *Candida* is known to cause food spoilage and produce unpleasant odors and flavors [42], this result highlighted the strong inhibitory effect of the combined treatment on spoilage-associated fungi.

In conclusion, the use of ultrasound with SAEW significantly curbed fungal growth, particularly the key spoilage genera, enhancing the quality and safety of duck breast meat throughout storage.

## 4. Conclusions

This research investigated the effects of combining ultrasound with SAEW on preserving duck breast meat during storage. The results demonstrated that this novel treatment significantly slowed the increase in pH values, reduced the generation of TVB-N, and effectively mitigated spoilage compared to untreated or individually treated samples. It also preserved superior color quality by maintaining the a* value, improved the meat’s texture, and delayed protein oxidation, as evidenced by the slower decline in total sulfhydryl content and reduced carbonyl levels. Additionally, the combined treatment exhibited potent antimicrobial effects, keeping the total viable count within safe limits by day 9 of storage and significantly suppressing spoilage-associated genera such as *Pseudomonas* and *Candida*. These findings highlight the potential of ultrasound combined with SAEW as an innovative preservation technique that enhances the storage quality, freshness, texture, and the nutritional value of duck breast meat while extending its shelf life. This method not only addresses the growing demand for improved food preservation but also offers opportunities for reducing food waste and enhancing the supply chain of fresh meat products. Future studies could explore optimizing this technique for other meat types, scaling up for industrial applications, and assessing its environmental and economic sustainability.

## Figures and Tables

**Table 1:** Changes in the color of duck breast meat during storage.

Storage Time (Days)	Group	L*	a*	b*
0	WD	47.98 ± 1.12 ^b^	19.51 ± 0.71 ^a^	8.44 ± 0.63 ^b^
	SD	48.73 ± 0.67 ^a^	19.09 ± 0.8 ^ab^	9.12 ± 1.51 ^a^
	UWD	48.75 ± 1.09 ^a^	19.2 ± 0.84 ^ab^	7.1 ± 0.49 ^c^
	USD	47.33 ± 1.65 ^c^	19.79 ± 0.49 ^a^	9.23 ± 1.19 ^a^
6	WD	49.01 ± 0.07 ^a^	17.06 ± 0.35 ^ab^	6.57 ± 1.31 ^c^
	SD	48.17 ± 0.3 ^b^	17.42 ± 0.49 ^a^	8.69 ± 0.17 ^a^
	UWD	45.31 ± 0.72 ^c^	17.21 ± 0.11 ^ab^	6.43 ± 0.63 ^c^
	USD	47.23 ± 0.62 ^c^	17.57 ± 0.03 ^a^	7.13 ± 0.44 ^b^
12	WD	49.9 ± 0.8 a^a^	16.07 ± 0.42 ^bc^	9.69 ± 0.28 ^a^
	SD	46.74 ± 0.15 ^c^	17.39 ± 0.29 ^a^	8.56 ± 0.2 ^b^
	UWD	47.54 ± 1.61 ^b^	15.49 ± 0.05 ^d^	7.54 ± 0.35 ^d^
	USD	49.57 ± 1.23 ^a^	16.32 ± 0.61 ^b^	8.16 ± 0.71 ^bc^

Explanatory note: WD: duck breast meat treated with pure water; SD: duck breast meat treated with SAEW; UWD: duck breast meat treated with ultrasound combined with pure water; USD: duck breast meat treated with ultrasound combined with SAEW. Different letters within the same column on the same day indicate statistically significant differences at a level of *p* < 0.05.

**Table 2:** Alpha diversity of duck breast meat bacteria during storage.

Storage Time (Days)	Group	Chao 1	Observed Species	Shannon	Simpson
0	WD	205.1	204.7	5.12	0.56
	SD	56.99	151.8	2.06	0.88
	UWD	53.69	56.8	3.94	0.71
	USD	32.03	32	1.88	0.94
6	WD	207.53	161.3	1.73	0.44
	SD	162.23	73	1.61	0.56
	UWD	100.9	206.4	1.79	0.53
	USD	73.11	99.8	2.78	0.67
12	WD	126.9	102.4	3.03	0.63
	SD	103.43	92	2.33	0.69
	UWD	108.26	107.1	2.36	0.64
	USD	93.01	126.7	2.2	0.73

Explanatory note: WD: duck breast meat treated with pure water; SD: duck breast meat treated with SAEW; UWD: duck breast meat treated with ultrasound combined with pure water; USD: duck breast meat treated with ultrasound combined with SAEW.

**Table 3:** Alpha diversity of duck breast meat fungi during storage.

Storage Time (Days)	Group	Chao 1	Observed Species	Shannon	Simpson
0	WD	41	41	3.79	0.89
	SD	54.1	53.6	2.65	0.76
	UWD	48	48	3.56	0.88
	USD	55	55	2.38	0.67
6	WD	55.01	54.9	3.59	0.81
	SD	42.31	42	3.2	0.81
	UWD	61.1	61	3.41	0.82
	USD	47.9	47.9	3.07	0.83
12	WD	45	45	3.54	0.85
	SD	47	46.9	3.05	0.81
	UWD	41.05	41	3.27	0.78
	USD	35	35	3.05	0.78

Explanatory note: WD: duck breast meat treated with pure water; SD: duck breast meat treated with SAEW; UWD: duck breast meat treated with ultrasound combined with pure water; USD: duck breast meat treated with ultrasound combined with SAEW.

## Data Availability

The raw data supporting the conclusions of this article will be made available by the authors on request. The data are not publicly available due to privacy restrictions.

## Supplementary Materials

The following supporting information can be downloaded at: https://www.mdpi.com/article/10.3390/foods14030534/s1.
